# Editorial: Regulation of proteostasis and cellular energy homeostasis at the primary cilium

**DOI:** 10.3389/fcell.2023.1285237

**Published:** 2023-09-08

**Authors:** Thorsten Pfirrmann, Brunella Franco, Daniel Kopinke, Christoph Gerhardt

**Affiliations:** ^1^ Department of Medicine, Institute for Molecular Medicine, Health and Medical University, Potsdam, Germany; ^2^ Telethon Institute of Genetics and Medicine (TIGEM), Federico II University of Naples, School for Advanced Studies, Genomics and Experimental Medicine Programme, Naples, Italy; ^3^ Department of Pharmacology and Therapeutics, Myology Institute, University of Florida, Gainesville, FL, United States

**Keywords:** primary cilia, proteostasis, energy homeostasis, signal transduction pathways, ciliopathies, autophagy, ubiquitin proteasome system

Primary cilia are fascinating, evolutionary conserved cell organelles involved in sensing of extracellular signals during development and adulthood and are consequently considered to function as the “antenna of the cell.” Morphologically, primary cilia also remind of an antenna and form a protrusion that sticks out of the cell. This protrusion consists of a nine-duplet microtubular filament ring that arises from the mother centriole and is covered by the ciliary membrane. The ciliary membrane contains various receptor proteins and consequently a growing number of signaling pathways originate at primary cilia. These comprise Hedgehog (HH) signaling, transforming growth factor (TGF)-β signaling, G protein-coupled receptor (GPCR) signaling, platelet-derived growth factor receptor (PDGFR)-α, mechanistic target of rapamycin (mTOR) signaling, Hippo signaling, Notch signaling, Wnt signaling and many more (Mill et al., 2023). Recent evidence even suggests an interplay between primary cilia and cellular protein homeostasis (proteostasis), a process defined as the fine-tuned balance between protein synthesis, protein folding and protein degradation through the ubiquitin proteasome system (UPS) and autophagy (Malicki and Johnson, 2017; Morleo et al., 2023). The posttranslational modification of proteins with ubiquitin (ubiquitination) is important for the regulation of proteostasis and effective polyubiquitination of proteins necessitates the creation of an isopeptide bond between the C-terminal glycine residue of ubiquitin and either a lysine residue within the substrate or within another ubiquitin molecule, e.g., lysine 48 (K48) (Kerscher et al., 2006). Several components of the ubiquitin proteasome system (UPS) including a specialized proteasome are present at the primary cilium (Ger et al., 2015; Habeck and Schweiggert, 2022; Hantel et al., 2022; Chiuso et al., 2023).

**FIGURE 1:**
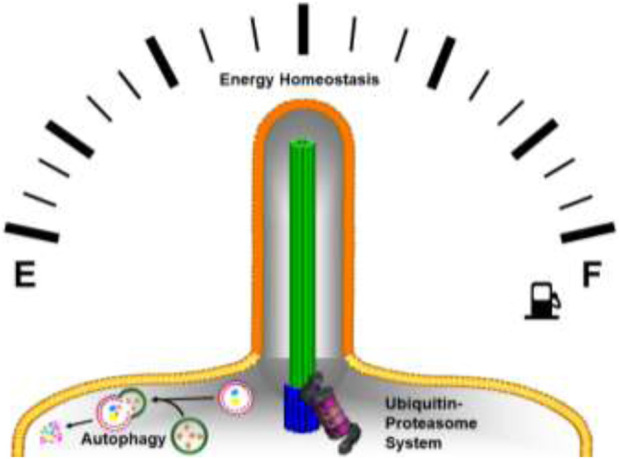
Cartoon of the primary cilium that functions as a fuel gauge to regulate energy homeostasis and proteostasis with the following color code; axoneme (green), basal body (blue), the ciliary membrane (orange), plasma membrane (yellow).

Primary cilia dysfunction plays a fundamental role in numerous hereditary organ-specific or syndromic diseases that are collectively called ciliopathies. Diseases and syndromes resulting from pathological changes in the primary cilium are diverse and occur in their entirety relatively frequent (1:2000) (Kagan et al., 2017). Similar to the diverse pathological changes, the clinical picture of ciliopathies is wide-ranging from isolated organ manifestations with restriction to the eye in Leber’s congenital amaurosis, to syndromic and severe diseases like the Meckel-Gruber syndrome, characterized by defects in the central nervous system (most frequently occipital encephalocele), postaxial polydactyly, cystic kidneys, cystic liver, ductal proliferation in the portal area of the liver, eye defects (e.g., microphthalmia), orofacial clefts and heart abnormalities (Reiter and Leroux, 2017). Certain ciliopathies, such as Bardet-Biedl syndrome (BBS, OMIM #209900) and Alström syndrome (ALMS, OMIM #203800), have been associated with obesity and metabolic disorders, suggesting that the primary cilium functions as a fuel gauge that regulates energy homeostasis and proteostasis (Figure 1) (Collin et al., 2002; Forsythe and Beales, 2013).

The relationships between primary cilia and proteostasis as well as between primary cilia and energy homeostasis are emerging fields in the scientific community and mechanisms that mediate primary cilia or basal body derived signals to modulate both processes are largely unknown. To bring together our current knowledge in the field, we present a compilation of four excellent review articles and two original research articles that contribute to shed light into this fascinating Research Topic.

Ubiquitination plays a crucial role in promoting substrate unfolding and subsequent degradation by the proteasome, making it the primary inducer of this process. However, to this end a comprehensive landscape of ubiquitinated proteins at the cilium is missing. Aslanyan et al. deploys a multi-proteomics approach using both ciliary-targeted ubiquitin affinity proteomics, as well as ubiquitin-binding domain-based proximity labelling to fill this gap and to create an overview of proteins modified with ubiquitin within the primary cilium. The authors identify several key proteins involved in signaling, cytoskeletal remodeling and protein trafficking including ESCRT-dependent clathrin-mediated endocytosis-related proteins and structural components of caveolae, e.g., CAV1, CAVIN1, EHD2.

Two specific ciliopathies, Ellis van Creveld syndrome (EvC) and Weyers acrofacial dysostosis (WAD), primarily affect skeletal development. Both are caused by mutations in genes encoding for proteins of the heterodimeric, ciliary transmembrane EVC-EVC2 complex that is involved in Hedgehog signaling. Despite the significance of this complex, it remains only poorly understood how the stability and targeting to the place of action within the cilium is mechanistically controlled. Barbeito et al. provide evidence, that ubiquitination of the complex could play a role in the regulation of complex stability. In line with this, the authors provide a list of EVC interacting proteins that includes the deubiquitinating enzyme USP7, suggesting that USP7 functions at the EVC-EVC2 complex. Furthermore, the authors identify previously unknown targeting signals in the C-terminus of EVC2 that are essential for the accumulation of the complex at the ciliary base.

Bardet-Biedl Syndrome (BBS, OMIM #209900) and Alström Syndrome (ALMS, OMIM #203800), have been linked to obesity and metabolic disorders. Recent studies emphasize the significant role of the primary cilium in maintaining whole-body energy homeostasis. This is achieved through ciliary signaling in various cell types and tissues relevant to metabolism. In a comprehensive review, Scamfer et al. provide an overview of the current knowledge regarding regulation of energy homeostasis by the cilium in adipose tissue and regarding ciliary proteins taking part in hypothalamic neuron signaling and adipocyte differentiation.

In a comprehensive review by Melena and Hughes the current understanding of how cilia contribute to islet hormone regulation and glucose homeostasis is consolidated. People with diabetes of all types experience glucose dysregulation due to changes in the function and coordination of pancreatic islet cells. Various events leading to hormone secretion to regulate these processes are precisely regulated at/by the primary cilium, among them ciliary pathways that govern insulin exocytosis and intercellular communication.

Brewer et al. provide a brief overview of syndromic ciliopathies and monogenic cilia signaling mutations related to obesity and focus on neuronal cilia to underscore the critical role of neuronal cilia-mediated signaling in maintaining proper energy homeostasis. Furthermore, the authors summarize literature on cilia and leptin-melanocortin signaling, as well as on changes in ciliary G protein-coupled receptor (GPCR) signaling and put a focus on brain regions where cilia are involved in energy homeostasis.

Claude-Taupin et al. explore the intricate cross-talk between autophagy, the primary cilium, and physical forces in the regulation of processes like mitochondrial biogenesis and lipophagy. Recent developments summarized in this review article unveil an interconnection between autophagy and the primary cilium, which affects autophagy but also contributes to the control of ciliogenesis.

In conclusion, this Research Topic includes six articles addressing novel and updated aspects related to the regulation of proteostasis and energy homeostasis at/by the primary cilium and will likely turn into a standard reference in the coming years.
